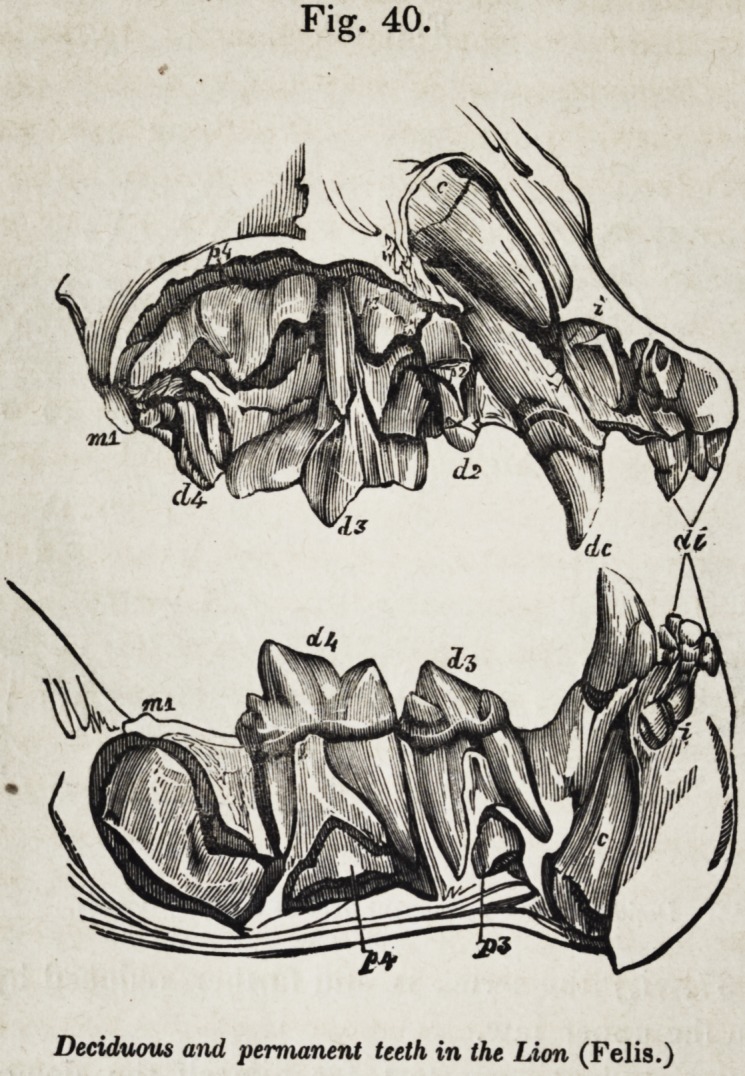# Teeth—Comparative Anatomy

**Published:** 1852-07

**Authors:** 

**Affiliations:** from the Cyclopœdia of Anatomy and Physiology


### SELECTED ARTICLES.

### ARTICLE XII.

### *Teeth—Comparative Anatomy.*

(Continued from page 489.)

The apex of the tooth soon begins to be worn away, and it would appear, by many specimens that have been found, that the teeth were retained until nearly the whole of the crown had yielded to the daily abrasion. In these teeth, however, the deep excavation of the remaining fang, represented in profile in fig. 27, plainly bespeaks the progress of the successional tooth prepared to supply the place of the worn-out grinder.

At the earlier stages of abrasion, a sharp edge is maintained at the external part of the tooth by means of the enamel which covers that surface of the crown. The prominent ridges upon that surface give a sinuous contour to the middle of the cutting edge, whilst its sides are jagged by the lateral serrations. The adaptation of this admirable dental instrument to the cropping and comminution of such tough vegetable food as the *clathrariæ* and similar plants, which are found buried with the iguanodon, is pointed out by Dr. Buckland, with his usual felicity of illustration, in his "Bridgewater Treatise," vol. i, p. 246.

When the crown is worn away beyond the enamel, it presents a broad and nearly horizontal grinding surface, and now another dental substance is brought into use to give an inequality to that surface; this is the ossified remnant of the pulp, which, being firmer than the surrounding dentine, forms a slight transverse ridge in the middle of the grinding surface. The tooth in this stage has exchanged the functions of an incisor for that of a molar, and is prepared to give the final compression, or comminution, to the coarsely divided vegetable matters.

The marginal edge of the incisive condition of the tooth and the median ridge of the molar stage are more effectually established by the introduction of a modification into the texture of the dentine, by which it is rendered softer than in the existing iguanæ and other reptiles, and more easily worn away: this is effected by an arrest of the calcifying process along certain cylindrical tracts of the pulp, which is thus continued, in the form of medullary canals, analogous to those in the soft dentine of the megatherium's grinder, from the central cavity, at pretty regular intervals, parallel with the calcigerous tubes, nearly to the surface of the tooth. The medullary canals radiate from the internal and lateral sides of the pulp-cavity, and are confined to the dentine forming the corresponding walls of the tooth; their diameter is $\frac{1}{1250}$th of an inch; they are separated by pretty regular intervals, equal to from six to eight of their own diameters; they sometimes divide once in their course. Each medullary canal is surrounded by a clear substance; its cavity was occupied in the section described by a substance of a deeper yellow color than the rest of the dentine.

The calcigerous tubes present a diameter of $\frac{1}{25000}$th of an inch, with interspaces equal to about four of their diameters. At the first part of their course, near the pulp-cavity, they are bent in strong undulations, but afterwards proceed in slight and regular primary curves, or in nearly straight lines, to the periphery of the tooth. When viewed in a longitudinal section of the tooth, the concavity of the primary curvature is turned towards the base of the tooth; the lowest tubes are inclined towards the root, the rest have a general direction at right angles to the axis of the tooth; the few calcigerous tubes, which proceed vertically to the apex, are soon worn away and can be seen only in a section of the apical part of the crown of an incompletely developed tooth. The secondary undulations of each tooth are regular and very minute. The branches, both primary and secondary, of the calcigerous tubes are sent off from the concave side of the main inflections; the minute secondary branches are remarkable at certain parts of the tooth for their flexuous ramifications, anastomoses, and dilatations into minute calcigerous cells, which take place along nearly parallel lines for a limited extent of the course of the main tubes. The appearance of interruption in the course of the calcigerous tubes, occasioned by this modification of their secondary branches, is represented by the irregularly dotted tracts in the figure of the dental structure of this ancient reptile given in my "Odontography." This modification must contribute, with the medullary canals, though in a minor degree, in producing that inequality of texture and of density in the dentine, which renders the broad and thick tooth of the iguanodon more efficient as a triturating instrument.

The enamel which invests the harder dentine, forming the outer side of the tooth, presents the same peculiar dirty brown color, when viewed by transmitted light, as in most other teeth: very minute and scarcely perceptible undulating fibres, running vertically to the surface of the tooth, is the only structure I have been able to detect in it.

The cement is simply and minutely cellular upon the crown of the tooth, but it exhibits the radiated cells at the base of the tooth.

The remains of the pulp in the contracted cavity of the completely formed tooth, are converted into a dense but true osseous substance, characterised by minute elliptical radiated cells, whose long axis is parallel with the plane of the concentric lamellæ, which surround the few, and contracted medullary canals in this substance.

The microscopical examination of the structure of the iguanodon's teeth, thus contributes additional evidence of the perfection of their adaptation to the offices to which their more obvious characters had indicated them to have been destined.

To preserve a trenchant edge, a partial coating of enamel is applied; and, that the thick body of the tooth might be worn away in a more regularly oblique plane, the dentine is rendered softer as it recedes from the enameled edge, by the simple contrivance of arresting the calcifying process along certain tracts of the inner wall of the tooth. When attrition has at length exhausted the enamel, and the tooth is limited to its functions as a grinder; a third substance has been prepared in the ossified remnant of the pulp, to add to the efficiency of the dental instrument in its final capacity. And if the following reflections were natural and just after a review of the externa lcharacters of the dental organs of the iguanodon, their truth and beauty become still more manifest, as our knowledge of their subject becomes more particular and exact:

"In this curious piece of animal mechanism, we find a varied adjustment of all parts and proportions of the tooth, to the exercise of peculiar functions, attended by compensations adapted to shifting conditions of the instrument, during different stages of its consumption. And we must estimate the works of nature, by a different standard from that which we apply to the productions of human art; if we can view such examples of mechanical contrivance, united with so much economy of expenditure, and with such anticipated adaptations to varying conditions in their application, without feeling a profound conviction, that all this adjustment has resulted from design and high intelligence."

*Varanians.*—In the great crocodilian monitor *(varanus crocodilinus,)* the large fixed compressed teeth, of which there may be about seven in each upper maxillary bone, and six in each premandibular, are anchylosed by the whole of their base and by an oblique surface leading upwards on the outer side of the tooth to a slight depression on the oblique alveolar surface, as in the *var. striatus.* The base of the tooth is finely striated, the lines being produced by inflected folds of the external cement, as in the ichthyosaur and labyrinthodon, but they are short and straight, as in those of the former genus. The alveolar channel or groove has scarcely any depth; but the anchylosed base of the tooth is applied to an oblique surface, terminating in a sharp edge, from which the outer side of the free crown of the tooth is directly continued. The great *varanus*, like the variegated species manifests its affinity to the crocodilians in the number of successive teeth which are in progress of growth to replace each other; but from the position in which the germs of the successional teeth are developed, the more advanced teeth in this species, as in the *var. variegatus*, do not exhibit the excavations that characterise the same parts of the teeth of the enaliosaurs and crocodiles.

*Thecodonts.*—We have seen, that among the inferior or squamate saurians, there are two leading modifications in the mode of attachment of the teeth ; the base of which, may be either anchylosed to the summit of an alveolar ridge, or to the bottom of an alveolar groove, and supported by its lateral wall. These modifications are indicated, respectively, by the terms "acrodont" and "pleurodont." A third mode of fixation is presented by some extinct saurians, which, in other parts of their organization, adhere to the squamate or lacertine division of the order, the teeth being implanted in sockets, either loosely or confluent with the bony walls of the cavity ; these I have termed the "thecodont" lacertians : the most ancient of all saurians belong to this group ; viz. the thuringian monitor, or *protorosaurus*, and the *palæosaurus* of the dolomitic conglomerates near Bristol. The compressed varanian form of tooth, with trenchant and finely dentated margins, which characterised the ancient palœosaur and chadeiodon, is continued in the comparatively more recent and gigantic species of terrestrial lizard, of which the remains were discovered by Dr. Buckland, in the oolite of Stonesfield, by whom, the peculiarities of the jaws and teeth have been accurately and graphically described, in the following words :

"From these remains, we learn, that the animal was a reptile, closely allied to some of our modern lizards ; and viewing the teeth as instruments for providing food to a carnivorous creature of enormous magnitude, they appear to have been admirably adapted to the destructive office for which they have been designed. Their form and mechanism will be best explained by reference to the figures.

The outer margin of the jaw rises nearly an inch above its inner margin, forming a continuous lateral parapet, to support the teeth on the exterior side, where the greatest support was necessary, whilst the inner margin throws up a series of triangular plates of bone forming a zigzag buttress along the interior of the alveoli. From the centre of each triangular plate, a bony partition crosses to the outer parapet, thus completing the successive alveoli. The new teeth are seen in the angle between each triangular plate, rising in reserve to supply the loss of older teeth, as often as progressive growth, or accidental fracture, may render such renewal necessary, and thus affording an exuberant provision for a rapid succession and restoration of these most essential implements. They were formed in distinct cavities, by the side of the old teeth, towards the interior surface of the jaw, and probably, expelled them by the usual process of pressure and absorption, insinuating themselves into the cavities thus left vacant. This contrivance for the renewal of teeth is strictly analogous to that which takes place in the dentition of many species of existing lizards.

In the structure of these teeth, we find a combination of mechanical contrivances analogous to those which are adopted in the construction of the knife, the sabre, and the saw. When first protruded above the gum, the apex of each tooth presented a double cutting edge of serrated enamel. In this stage, its position and line of action were nearly vertical, and its form, like that of the two-edged point of a sabre, cutting equally on each side. As the tooth advanced in growth, it became curved backwards in the form of a pruning-knife, and the edge of serrated enamel was continued downwards to the base of the inner and cutting side of the tooth, whilst on the outer side, a similar edge descended but a short distance from the point, and the convex portion of the tooth became blunt and thick, as the back of a knife is made thick for the purpose of producing strength. The strength of the tooth was further increased by the expansion of its side. Had the serrature continued along the whole of the blunt and convex portion of the tooth, it would, in this position, have possessed no useful cutting power; it ceased precisely at the point beyond which it could no longer be effective. In a tooth thus formed for cutting along its concave edge, each movement of the jaw combined the power of the knife and saw; whilst the apex, in making the first incision, acted like the two-edged point of a sabre. The backward curvature of the full grown teeth, enabled them to retain, like barbs; the prey which they had penetrated. In these adaptations, we see contrivances which human ingenuity has also adopted in the preparation of various instruments of art.

The teeth of the *megalosaur* consist of a central body of dentine, with an investment of enamel upon the crown, and of cement over all, but thickest upon the fang. The marginal serrations are formed almost entirely by the enamel, and when slightly magnified, are seen to be rounded, and separated by slight basal grooves; the smooth and polished enamel upon the sides of the crown, presents a finely wrinkled appearance; the remains of the pulp are converted into a coarse bone in the completely formed tooth.

*Enaliosaurs.*—The teeth of the ichthyosauri have a simple, more or less acutely conical form, with a long and, usually, expanded or ventricose base, or implanted fang. They are confined to the intermaxillary, maxillary, and premandibular bones, in which they are arranged in a pretty close and uninterrupted series, and are of nearly equal size. They consist of a body of unvascular dentine, invested at the base by a thick layer of cement, and at the crown by a layer of enamel, which is itself covered by a very thin coat of cement; the pulp-cavity is more or less occupied in fully-formed teeth by a coarse bone. The external surface of the tooth is marked by the longitudinal impressions and ridges, but the teeth vary both as to outward sculpturing and general form in the different species.

The chief peculiarity of the dental system of the ichthyosaur, is the mode of the implantation of the teeth; instead of being anchylosed to the bottom and side of a continuous shallow groove, as in most lacertians, are implanted in distinct sockets, as in the thecodon, megalosaur, or pterodactyle, they are lodged loosely in a long and deep continuous furrow, and retained by slight ridges between the teeth, along the sides and bottom of the furrow, and by the gum and organized membranes, continued into the groove and upon the base of the teeth.

The germs of the new teeth are developed at the inner side of the base of the old ones.

*Crocodilia.*—The best and most readily recognizable characters, by which the existing crocodilians are grouped in appropriate genera, are derived from modifications of the dental system.

In the caimans, (genus *alligator*,) the teeth vary in number from $\frac{18}{18}$—$\frac{18}{18}$ to $\frac{22}{22}$—$\frac{22}{22}$: the fourth tooth of the lower jaw, or canine, is *received into a cavity* of the palatal surface of the upper jaw, where it is concealed when the mouth is shut. In old individuals, the upper jaw is perforated by these large inferior canines, and the fossæ are converted into foramina.

In the crocodiles, (genus *crocodilus*,) the first tooth in the lower jaw perforates the palatal process of the premaxillary bone when the mouth is closed; the fourth tooth in the lower jaw is *received into a notch* excavated in the side of the alveolar border of the upper jaw, and is visible externally when the mouth is closed.

In the two preceding genera, the alveolar borders of the jaw have an uneven or wavy contour, and the teeth are of an unequal size.

In the gavials, (genus *gavialis*,) the teeth are nearly equal in size, and similar in form, in both jaws, and the first, as well as the fourth tooth in the lower jaw, passes into a groove in the margin of the upper jaw when the mouth is closed.

In the alligators and crocodiles, the teeth are more unequal in size, and less regular in arrangement, and more diversified in form than in the gavials: witness the strong thick conical laniary teeth as contrasted with the blunt mammillate summits of the posterior teeth in the alligator (fig. 28.) The teeth of the gavial are subequal, most of them present the form of crown, shown in fig. 29, long, slender, pointed, subcompressed from before backwards, with a trenchant edge on the right and left sides, between which a few faint longitudinal ridges traverse the basal part of the enamelled crown.

Fig. 29

Amongst the remains of crocodilians which are scattered through the tilgate strata, the most common ones are detached teeth, from the difference observable, in the form of which, Dr. Mantell has observed, that "they appear referable to two kinds, the one belonging to that division of crocodiles, with long slender muzzles, named *gavial*, the other to a species of *crocodile*, properly so-called, and resembling a fossil species found at Caen."

Dr. Mantell has obligingly communicated to me figures of well-preserved specimens of both the forms of teeth alluded to, the exactness of which I have recognized by a comparison with the specimens, themselves, in the British Museum.

*Teeth in different stages of formation from one alveolus of the gavial:* ***a*** is the base partly absorbed by the pressure of *b*, the successional tooth; below which is figured *c*, the germ of the next tooth to follow.

The tooth, which, from its more slender and acuminated form, approaches nearest to the character of those of the gavial, presents a marked difference, however, from the teeth of any of the recent species of that sub-genus, the crocodilians, as well as from those of the long and slender-snouted extinct genera, called *teleosaurus*, *stineosaurus*, &c. I have described it, therefore, as indicative of a distinct species, under the name of *crocodilus cultridens.* The crown is laterally compressed, subincurved, with two opposite trenchant edges, one forming the concave, the other the convex, outline of the tooth. In the gavial, the direction of the flattening of the crown, and the situation of the trenchant edges are the reverse, the compression being from before backwards, and the edges being lateral.

The tooth of the *crocodilus cultridens*, thus resembles, in form, that of the megalosaur, and perhaps still more those of the argenton crocodile; but I have not observed any specimens of the wealden teeth in which the edges of the crown were serrated, as in both the reptiles just cited. The teeth of the *crocodilus cultridens*, also present a character which does not exist in the teeth of the megalosaur, and is not attributed by Cuvier to those of the *crocodile d'argenton.* The sides of the crown are traversed by a few longitudinal parallel ridges, with regular intervals of about one line, in a crown of a tooth one inch and a half in length: these ridges subside before they reach the apex of the tooth, and more rapidly at the convex than at the concave side of the crown.

Hitherto these teeth have not been found so associated with any part of the skeleton of the same species as to yield further characters of the present extinct crocodilian; but from the above-mentioned well-marked differences between these teeth and those of all the existing species, it is most probable that the extinct crocodile formed the type of a distinct sub-genus, for which the term *suchosaurus* has been proposed.

The second form of tooth having the generic characters of those of the crocodile, which has been discovered in the wealden and approximate strata, is as remarkable for its thick, rounded, and obtuse crown, as the teeth of the preceding species are for their slender, compressed, acute, and trenchant character. It consequently approaches more nearly to the teeth, which characterize the broad and comparatively short-snouted crocodiles; but it differs from these in one of the same characters by which the tooth of the *suchosaurus cultridens* differs from those of the gavials, viz. in the longitudinal ridges which traverse the exterior of the crown. These are, however, more numerous, more close-set, and more neatly defined than in the *suchosaurus cultridens.* Two of the ridges, larger and sharper than the rest, traverse opposite sides of the tooth, from the base to the apex of the crown; they are placed, as in the crocodile and gavial, at the sides of the crown, midway between the convex and concave lines of the curvature of the tooth.

These ridges are confined to the enamel; the cement-covered cylindrical base of the tooth is smooth. The size of the teeth varies from a length of crown of two inches, with a basal diameter of one inch and a half to teeth of one-third of these dimensions. I have proposed to call this extinct crocodile, with biconcave vertebræ, *goniopholis crassidens.*

*Development.*—In the black alligator of Guiana, the first fourteen teeth of the lower jaw are implanted in distinct sockets, the remaining posterior teeth are lodged close together in a continuous groove, in which the divisions for sockets are faintly indicated by vertical ridges, as in the jaws of ichthyosaurs. A thin compact floor of bone separates this groove, and the sockets anterior to it, from the large cavity of the ramus of the jaw; it is pierced by blood-vessels for the supply of the pulps of the growing teeth, and the vascular dentiparous membrane which lines the alveolar cavities.

Fig. 30.

*Section of lower jaw, with four alveoli and teeth of the black alligator.*

The tooth-germ is developed from the membrane, covering the angle between the floor and the inner wall of the socket. It becomes, in this situation, completely enveloped by its capsule, and an enamel-organ is formed at the inner surface of the capsule before the young tooth penetrates the interior of the pulp-cavity of its predecessor.

The matrix of the young growing tooth affects, by its pressure, the inner wall of the socket, as shown in fig. 30, and forms for itself a shallow recess: at the same time it attacks the side of the base of the contained tooth; then, gaining a more extensive attachment by its basis and increased size, it penetrates the large pulp-cavity of the previously formed tooth, either by a circular or semi-circular perforation. The size of the calcified part of the tooth-matrix, which has produced the corresponding absorption of the previously formed tooth on the one side, and of the alveolar process on the other, is represented in the second exposed alveolus of fig. 30, the tooth *a* having been displaced and turned round to show the effects of the stimulus of the pressure. The size of the perforation in the tooth, and of the depression in the jaw, proves them to have been, in great part, caused by the soft matrix, which must have produced its effect by exciting vital action of the absorbents, and not by mere mechanical force. The resistance of the wall of the pulp-cavity having been thus overcome, the growing tooth and its matrix recede from the temporary alveolar depression, and sink into the substance of the pulp contained in the cavity of the fully-formed tooth. As the new tooth grows, the pulp of the old one is removed; the old tooth itself is next attacked, and the crown being undermined by the absorption of the inner surface of its base, may be broken off by a slight external force, when the point of the new tooth is exposed, as in the fig. 30, *b*.

The new tooth disembarrasses itself of the cylindrical base of its predecessor, with which it is sheathed, by maintaining the excitement of the absorbent process so long as the cement of the old fang retains any vital connection with the periosteum of the socket; but the frail remains of the old cylinder, thus reduced, are sometimes lifted off the socket upon the crown of the new tooth, as in fig. 30. *b*, when they are speedily removed by the action of the jaws. This is, however, the only part of the process which is immediately produced by mechanical force: an attentive observation of the more important previous stages of growth, teaches that the pressure of the growing tooth operates upon the one to be displaced only through the medium of the vital absorbent action which it has excited.

Most of the stages in the development and succession of the teeth of the crocodiles are described by Cuvier with his wonted clearness and accuracy; but the mechanical explanation of the expulsion of the old tooth, which Cuvier adopts from M. Tenon, is opposed by the disproportionate smallness of the hard part of the new tooth to the vacuity in the old one, and by the fact that the matter impressing, viz. the uncalcified part of the walls of the tooth-matrix—is less dense than the part impressed.

No sooner has the young tooth penetrated the interior of the old one, than another germ begins to be developed from the angle between the base of the young tooth and the inner alveolar process, or in the same relative position as that in which its immediate predecessor began to rise, and the processes of succession and displacement are carried on, uninterruptedly, throughout the long life of these cold-blooded carnivorous reptiles.

From the period of exclusion from the egg, the teeth of the crocodile succeed each other in the vertical direction; none are added from behind forwards, like the true molars in mammalia. It follows, therefore, that the number of the teeth of the crocodile is as great when it first sees the light as when it has acquired its full size; and, owing to the rapidity of the succession, the cavity at the base of the fully-formed tooth is never consolidated.

The fossil jaws of the extinct crocodilians demonstrate that the same law regulated the succession of the teeth, at the ancient epoch when those highly organised reptiles prevailed in greatest numbers, and under the most varied generic and specific modifications, as at the present period, when they are reduced to a single family, composed of so few and slightly varied species as to have constituted in the system of Linnæus a small fraction of his genius *lacerta.*

*Dental System of Mammals.*—The class mammalia, like that of *reptilia* and *pisces*, includes a few genera and species that are devoid of teeth: the true ant-eaters, *(myrmecophaga,)* the scaly ant-eaters, or pangolins, *(manis,)* and the spiny monotrematous ant-eater, *(echidna,)* are examples of strictly edentulous mammals. The *ornithorhynchus* has horny teeth, and the whales *(balæna* and *balænoptera)* have transitory embryonic calcified teeth, succeeded by whalebone substitute in the upper jaw. Horny processes analogous to, perhaps homologous with, the lingual and palatal teeth in fishes, are present in the *echidna.*

The female narwhal seems to be edentulous, but has the germs of two tusks in the substance of the upper jaw-bones; one of these becomes developed into a large and conspicuous weapon in the male narwhal, and accordingly, suggested to Linnæus, the name for its genus, of *monodon*, meaning single tooth; but the tusk is never median, like the truly single tooth on the palate of the myxine; and occasionally both tusks are developed in the narwhal. In another cetacean, the great bottle-nose, or *hyperoodon*, the teeth are reduced in the adult to two in number, whence the specific name *H. bidens*, but they are confined to the lower jaw. The sharp-nosed dolphin (*ziphius*) has also but two teeth, one in each ramus of the lower jaw; and this is, perhaps, a sexual character. The *delphinus griseus* has five teeth on each side of the lower jaw; but they soon become reduced to two Amongst the marsupial animals, the genus *tarsipes* is remarkable for the paucity as well as minuteness of its teeth.

The elephant has never more than one entire molar, or parts of two, in use on each side of the upper and lower jaws; to which are added two tusks, more or less developed in the upper jaw.

Some rodents, as the Australian water-rats, (*hydromys*,) have two grinders on each side of both jaws; which, added to the four cutting teeth in front, make twelve in all: the common number of teeth in this order is twenty; but the hares and rabbits have twenty-eight teeth. The sloth has eighteen teeth.

The number of teeth, thirty-two, which characterises man, the apes of the old world, and the true ruminants, is the average one of the class mammalia; but the typical number is forty-four.

The examples of excessive number of teeth are presented, in the order *bruta*, by the priodont armadillo, which has ninety-eight teeth; and, in the cetaceous order, by the cachalot, which has upwards of sixty teeth, though most of them are confined to the lower jaw; by the common porpoise, which has between eighty and ninety teeth; by the gangetic dolphin, which has one hundred and twenty teeth; and by the true dolphins, (*delphinus*,) which has from one hundred to one hundred and ninety teeth, yielding the maximum number in the class mammalia.

*Form.*—Where the teeth are in excessive number, as in the species above cited, they are small, equal, or sub-equal, and of a simple conical form; pointed, and slightly recurved in the common dolphin; with a broad and flattened base in the gangetic dolphin, (*inia ;*) with the crown compressed, and broadest in the porpoise; compressed, but truncate, and equal with the fang, in the priodon. The compressed triangular teeth become coarsely notched or dentated, at the hinder part of the series, in the great extinct cetaceous *zeuglodon.* The simple dentition of the smaller armadillos, of the orycterope, and of the three-toed sloth, presents a difference in the size, but little variety in the shape of the teeth, which are subcylindrical, with broad triturating surfaces; in the two-toed sloth, the two anterior teeth of the upper jaw are longer and larger than the rest, and adapted for piercing and tearing.

In almost all the other mammalia, particular teeth have special forms for special uses: thus, the front teeth, from being commonly adapted to effect the first coarse division of the food, have been called cutters or *incisors ;* and the back teeth, which complete its comminution, grinders, or *molars*; large conical teeth, situated behind the incisors, and adapted by being nearer the insertion of the biting muscles, to act with greater force, are called holders, tearers, laniaries, or more commonly *canine* teeth, from being well developed in the dog and other carnivoro, although they are given, likewise, to many vegetable feeders for defence or combat: *e. g.* musk-deer, (fig. 36, vii.) Molar teeth, which are adapted for mastication, have either tuberculate, or ridged, or flat summits; and usually are either surrounded by a fence of enamel, or are traversed by enamel plates arranged in various patterns. Certain molars in the dugong, the mylodon, and the zeuglodon, are so deeply indented laterally by opposite longitudinal grooves, as to appear, when abraded, to be composed of two cylindrical teeth cemented together, and the transverse section of the crown is bilobed. The teeth of the *glyptodon* were fluted by two analogous grooves on each side. The large molars of the capybara and elephant have the crown cleft into a numerous series of compressed transverse plates, cemented together side by side.

The teeth of the mammalia have usually so much more definite and complex a form than those of fishes and reptiles, that three parts are recognised in them: viz. the "fang," the "neck," and the "crown." The fang or root (*radix*) is the inserted part; the crown (*corona*) the exposed part; and the construction which divides these is called the neck (*cervix.*) The term "fang" is properly given only to the implanted part of a tooth of restricted growth, which fang gradually tapers to its extremity; those teeth which grow uninterruptedly have not their exposed part separated by a neck from their implanted part, and this generally maintains to its extremity the same shape and size as the exposed crown.

It is peculiar to the class mammalia to have teeth implanted in sockets by two or more fangs; but this can only happen to teeth of limited growth, and generally characterises the molars and premolars; perpetually growing teeth require the base to be kept simple, and widely excavated for the persisting pulp. In no mammiferous animal does anchylosis of the tooth with the jaw constitute a normal mode of attachment. Each tooth has its particular socket, to which it firmly adheres by the close co-adaptation of their opposed surfaces, and by the firm adhesion of the alveolar periosteum to the organised cement which invests the fang or fangs of the tooth; but in some of the cetacea, at the posterior part of the dental series, the sockets are wide and shallow, and the teeth adhere more strongly to the gum than to the periosteum ; in the cachalot, I have seen all the teeth brought away with the ligamentous gum, when it has been stript from the sockets of the lower jaw.

Teeth are fixed as a general rule in all vertebrata, and the only known exceptions are those presented by certain species of fishes, *e. g.* the sharks, lophioids, goniodonts. In the higher vertebrata, the movements of the teeth depend on those of the jaw bones to which they are affixed, but appear to be independent in the ratio of the size of the tooth to the bone to which it is attached. Thus, the extent of rotatory movement to which the large perforated poison fangs of the rattlesnake are subject, depends upon the rotation of the small maxillary bone. So, likewise, the seemingly individual movements of divarication and approximation observable in the large lower incisors of the *bathyergus* and *macropus*, are due entirely to the yielding nature of the symphysis uniting the two rami of the lower jaw in which those incisors are deeply and firmly implanted. It is no more a property of the teeth themselves than is that alternate removal of the lower teeth from, and bringing of them in contact with the upper teeth of the mouth, which one sees or feels in the act of mastication.

True teeth implanted in sockets, are confined in the mammalian class, to the maxillary, pre-maxillary, and mandibular, or lower maxillary bones, and form a single row in each. They may project only from the pre-maxillary bones, as in the narwhal, or only from the lower maxillary bone, as in ziphius ; or be apparent only in the lower maxillary bone, as in the cachalot ; or be limited to the superior and inferior maxillaries, and not present in the premaxillaries, as in the true *pecora*, and most *bruta* of Linnæus ; in general, teeth are situated in all the bones above mentioned. In man, where the premaxillaries early coalesce with the maxillary bones, where the jaws are very short, and the crowns of the teeth are of equal length, there is no interspace or "diastema" in the dental series of either jaw, and the teeth derive some additional fixity by their close apposition and mutual pressure. No inferior mammal now presents this character; but its importance, as associated with the peculiar attributes of the human organization, has been somewhat diminished by the discovery of a like contiguous arrangement of the teeth in the jaws of a few extinct quadrupeds: e. g. *anoplotherium*, *nesodon* and *dichodon*.

The teeth of the mammalia usually consist of hard unvascular dentine, defended at the crown by an investment of enamel, and everywhere surrounded by a coat of cement. The coronal cement is of extreme tenuity in man, quadrumana, and terrestrial carnivora; it is thicker in the herbivora, especially in the complex grinders of the elephant; and is thickest in the teeth of the sloths, megatherioids, dugong, walrus and cachalot. Vertical folds of enamel and cement penetrate the crown of the tooth in the ruminants, and in most rodents and pachyderms, characterises by their various forms the genera of the last two orders; but these folds never converge from equidistant points of the circumference of the crown towards its centre. The teeth of the quadrupeds of the order *bruta (edentata*, Cuv.*)* have no true enamel; this is absent likewise in the molars of the dugong and the cachalot. The tusks of the narwhal, walrus, dinotherium, mastodon and elephant, consist of modified dentine, which, in the last two great proboscidian animals, is properly called "ivory," and is covered by cement.

In the subjoined magnified view of a section of the molar of a megatherium, *t* is the hard dentine, *v* the vaso-dentine, and *c* the cement (fig. 31.)

The teeth in the mammalia, as in the aforegoing classes, are formed by superaddition of the hardening salts to pre-existing moulds of animal pulp or membrane, organised so as to insure the arrangement of the earthy particles according to that pattern which characterizes each constituent texture of the tooth.

The complexity of the primordial basis or matrix, corresponds, therefore, with that of the fully formed tooth, and is least remarkable in those conical teeth which consist only of dentine and cement. The primary pulp, which first appears as a papilla rising from the free surface of the alveolar gum, is the part of the matrix which by its calcification constitutes the dentine; it sinks into a cell, and becomes surrounded by a

Fig. 31.

closed capsule in every mammiferous species, at an early stage of the formation of the tooth; and, as the cement is the result of the ossification of the capsule, every tooth must be covered by a layer of that substance. In those teeth which possess enamel, the mould or pulp of that constituent, is developed from the capsule covering the coronal part of the dentinal pulp. In the simple teeth, the secondary or enamel pulp covers the crown like a cap; in the complex teeth, it sends processes into depressions of the crown, which vary in depth, breadth, direction, and number in the numerous groups of the herbivorous and omnivorous quadrupeds. The dentinal pulp, thus penetrated, offers corresponding complications of form; and as the capsule follows the enamel pulp in all its folds and processes, the external cavities or interspaces of the dentine become occupied by enamel and cement; the cement, like the capsule which formed it, being the outermost substance, and the enamel being interposed between it and the dentine. The dental matrix presents the most extensive interdigitation of the dentinal and enamel pulps in the capybara and elephant.

The matrix of the mammalian tooth sinks into a furrow and and soon becomes inclosed in a cell in the substance of the jaw bone, from which the crown of the growing tooth extricates itself by exciting the absorbent process, whilst the cell is deepened by the same process, and by the growth of the jaw into an alveolus for the root of the tooth. Where the formative parts of the tooth are re-produced indefinitely to repair by their progressive calcification the waste to which the working surface of the crown of the tooth has been subject, the alveolus is of unusual depth, and of the same form and diameter throughout, except in the immature animal, when it widens to its bottom or base. In teeth of limited growth, the dentinal pulp is reproduced in progressively decreasing quantity after the completion of the exterior wall of the crown, and forms by its calcification one or more roots or fangs, which taper more or less rapidly to their free extremity. The alveolus is closely moulded upon the implanted part of the tooth ; and it is worthy of special remark, that the complicated form of socket which results from the development of two or more fangs, is peculiar to animals of the class mammalia.

In the formation of a single fang, the activity of the re-productive process becomes enfeebled at the circumference, and is progressively contracted within narrower limits in relation to a single centre, until it ceases at the completion of the apex of the fang; which, though for a long time perforated for the admission of the vessels, and nerves to the interior of the tooth, is, in many cases, finally closed by the ossification of the remaining part of the capsule.

When a tooth is destined to be implanted by two or more fangs, the re-production of the pulp is restricted to two or more parts of the base of the coronal portion of the pulp, around the centre of which parts the sphere of its re-productive activity is progressively contracted. The intervening parts of the base of the coronal pulp adhere to the capsule, which is simultaneously calcified with them, covering those parts of the base of the crown of the tooth with a layer of cement. The ossification of the surrounding jaw being governed by the changes in the soft, but highly organized, dental matrix, fills up the spaces unoccupied by the contracted and divided pulp, and affords, by its periosteum, a surface for the adhesion of the cement or ossified capsule covering the completed part of the tooth.

The matrix of certain teeth does not give rise during any period of their formation to the germ of a second tooth, destined to succeed the first; this, therefore, when completed and worn down, is not replaced: all the true cetacea are limited to this simple provision of teeth. In the armadillos, megatherioids, and sloths, the want of germinative power, as it may be called, in the matrix, is compensated by the persistence of the matrix, and by the uninterrupted growth of the teeth.

In most other mammalia, the matrix of the first developed tooth gives origin to the germ of a second tooth, which sometimes diplaces, sometimes takes its place by the side of its predecessor and parent. All those teeth which are displaced by their progeny, are called temporary, deciduous, or milk teeth; the mode and direction in which they are displaced and succeeded, viz. from above downwards in the upper, from below upwards in the lower jaw; in both jaws vertically—are the same as in the crocodile; but the process is never repeated more than once in any mammiferous animal. A considerable proportion of the dental series is thus changed; the second, or permanent teeth, having a size and form as suitable to the jaws of the adult, as the displaced temporary teeth were adapted to those of the young animal. The permanent teeth, which assume places not previously occupied by deciduous ones, are always the most posterior in their position, and generally the most complex in their form. The successors of the deciduous incisors and canines differ from them chiefly in size; the successors of the deciduous molars may differ likewise in shape, in which case they have always less complex crowns than their predecessors.

The "bicuspids," in human anatomy, and the corresponding teeth, called "premolars," in the lower mammals, illustrate this law.

The first true molar owes the germ of its matrix to a vegetation or bud, separated by the fissiparous process from the matrix of the last deciduous tooth; but the backward elongation of the jaw affords space for its development by the side of its progenitor, during which process it may, in like manner, give origin to a second, and this to a third molar, succeeding each other from before backwards or horizontally.

In this successive germ-production, we find repeated the multiparous property of the dental matrix of the crocodile; but the concomitant growth of the jaw allows the second, third, and sometimes fourth generation of true molars to co-exist, and come into place side by side. In the unguiculate, and most of the ungulate, species of the placental division of the mammalian class, the fissiparous reproduction of horizontally succeeding teeth, stops at the third generation; in other words, they have not more than three true molars on each side of the upper and lower jaws. In the marsupial series, the same process extends to a fourth generation of true or horizontally succeeding molars; and in most of the species, the four true molars are in use and place at the same time; but in certain kangaroos, the anterior ones are shed before the posterior ones are developed. This successive decadence is still more characteristic of the grinding teeth of the elephant, which are finally reduced to a single molar tooth on each side of both jaws.

Thus the class mammalia, in regard to the times of formation and the succession of the teeth, may be divided into two groups:—the "*monophyodonts*," or those that generate a single set of teeth; and the "*diphyodonts*," or those that generate two sets of teeth.

The monophyodonts include the orders *monotremata*, *bruta*, (*edentata*, Cuv.,) and (*cetacea vera*, Cuv.:) all the rest of the order are diphyodonts. In these, the first set of teeth are called the milk or deciduous teeth: the second set, the adult or permanent teeth; although the teeth of this set are, for the most part, like those of the first set, of limited growth, contracting to a root or roots, and being shed in greater or less proportion during the life-time of the species; which life-time, in wild carnivora and herbivora, is dependent on, and would seem, indeed, to be determined by, the duration of the adult teeth.

Examples of some of the striking modifications of dental structure, presented by recent or extinct animals of the order,

*bruta*, are given in figs. 6, and 31, of the present article. It will be observed that I have qualified the generalization as regards the monophyodont character of the *cetacea*, by citing only that part of Cuvier's order which he termed "true or carnivorous cetacea." The animals of the order *sirenia*, (herbivorous cetacea of Cuvier,) differ in many organic particulars from the *cetacea* proper, and in none, perhaps, more strikingly than in having both deciduous and permanent teeth; this succession takes place, at least, with regard to the upper incisors of the *dugong*, fig. 32.

Fig. 32.

*Dentition of the Dugong* (Halicore indicus.)

These teeth project from the gum in the male sex; but neither upper nor lower incisors are visible in the female. The superior incisors are but two in number, in both sexes; in the male, they are moderately long, subtriedral, slightly and equally curved, of the same diameter from the base, and deeply excavated to near the apex, which is obliquely beveled off to a sharp edge, like the scalpriform teeth of the *rodentia*. When fully developed, only the extremity of the tusk projects from the jaw, at least seven-eighths of its extent being lodged in the socket, the parietes of which are entire; and the exterior of the great premaxillary bones presents an unbroken surface. In the female dugong, the growth of the permanent incisive tusks of the upper jaw is arrested before they cut the gum, and they remain, through life, concealed in the premaxillaries; the tusk is solid, is about an inch shorter and less bent than that of the male; it is also irregularly cylindrical, longitudinally indented, and it gradually diminishes to an obtuse rugged point; the base is suddenly expanded, bent obliquely outwards, and presents a shallow excavation. These were conjectured, by Home, to be the "milk-tusks;" they are, however, characteristic of sex, not of age; and the existence of deciduous tusks at any period in the dugong has been called in question. I have, however, discovered in specimens of the Malayan dugong, which I have dissected at the Zoological Society, the true deciduous incisors of the upper jaw (fig. 32, *d i*) co-existing with the permanent ones (*i.*) They are much smaller than the permanent tusks of the female, and are loosely inserted by one extremity, in conical sockets, immediately anterior to those of the permanent tusks, adhering, by their opposite ends, to the thick, tegumentary gum, which presented no outward indication of their presence.

When this gum was stripped off the bone, the deciduous tusks came away with it; and this may account for their usual absence in dried crania of immature dugongs, in which, nevertheless, their alveoli are generally sufficiently conspicuous. True permanent incisors are not developed in the lower jaw of the dugong; those which are occasionally found there, are abortive remnants of the first, or deciduous series, which are not destined at any time to rise above the gum (fig. 32, *d i* 3.)

The molar teeth of the dugong resemble those of the order *bruta*, in the total absence of enamel, and of any constriction defining the crown from the fangs. In the Malayan species, only five molars (fig. 32, 1, 2, 3, 4, 5,) are developed on each side of both jaws: in the Australian dugong, six are developed; *i. e.* the *halicore indicus* is characterized by the molar formula *m.* $\frac{5-5}{5-5}=20$, whilst the *halicore australis* has *m.* $\frac{6-6}{6-6}=24$. But in both species, the number is progressively reduced, by the shedding of the anterior and smaller molars, to *m.* $\frac{2-2}{2-2}=8$. The structure of these molar teeth is illustrated in fig. 4, B, their form in fig. 4, A; the last molar, when it comes into use, presents a bilobed form of grinding surface, as is shown at *b*, fig. 32.

Owing to there being but one set of molars in the dugong, those teeth cannot be divided into true and false molars, any more than in the sloths or armadillos. In the true diphyodonts, in which each kind of teeth have deciduous predecessors, those grinders which succeed the deciduous ones vertically, and displace them, are called "premolars," or "false molars," and those that come into place behind these, without pushing out vertically any predecessors, are the "molar proper," or "true molars." In this article, the two sorts of grinders are called respectively "premolars" and "molars." In the marsupial order the normal number of molars is four in each dental series, *i. e. m.* $\frac{4-4}{4-4}$; in the placental diphyodonts there normal number is three, *i. e. m.* $\frac{3-3}{3-3}$; the normal number of premolars in the marsupialia is $\frac{3-3}{3-3}$, but in the placentalia, it is $\frac{4-4}{4-4}$: in both the numerical character of the canines is one, *i. e.* $\frac{1-1}{1-1}$; that of the incisors three, *i. e.* $\frac{3-3}{3-3}$. As regards the latter teeth, however the number of exceptions in the *marsupialia* is considerable, and the incisors are sometimes in excess; whilst in the placental diphyodonts, the incisors never exceed the typical number, but frequently depart from it by suppression or arrest of development.

In fishes and reptiles, certain teeth might be called "incisive," "laniary," or "molar" teeth, in reference to the special adaptation of their form for cutting, tearing, or bruising; but such terms, in the cold-blooded classes, imply nothing more than those modifications of form; they are not significative of constant and well defined groups of teeth, and could not become the names of definite parts or organs determinable and traceable from one species to another. In the mammalian orders, with two sets of teeth, these organs acquire fixed individual characters, receive special denominations, and can be determined from species to species. This individualization of the teeth is eminently significative of the high grade of organization of the animals manifesting it; especially when we consider the great proportion of mineral substance which enters into the composition of those parts; in the number and nature of which the principle of vegetative repetition, and the power of the general polarising forces, have been most controlled in the mammalia.

Originally, indeed, the name "incisors," "laniaries," or "canines," and "molars" were given to the teeth, in man and certain mammals, as in reptiles, in reference merely to the shape and offices so indicated; but they are now used as arbitrary signs, in a more fixed and determinate sense. In some carnivora, *e. g.*, the front teeth have broad tuberculate summits, adapted for nipping and bruising, while the principal back teeth are shaped for cutting, and work upon each other like the blades of scissors. The front teeth in the elephant project from the upper jaw, in the form, size, and direction of long pointed horns. In short, shape and size are the least constant of dental characters in the mammalia: and the homologous teeth are determined, like other parts, by their relative position, by their connections, and by their development.

Those teeth which are implanted in the premaxillary bones, and in the corresponding part of the lower jaw, are called "incisors," whatever be their shape or size. The tooth in the maxillary bone, which is situated at, or near to, the suture with the premaxillary, is the "canine," as is also that tooth in the lower jaw which, in opposing it, passes in front of its crown when the mouth is closed. The other teeth of the first set are the "deciduous molars:" the teeth which displace and succeed them vertically are the "premolars;" the more posterior teeth, which are not displaced by vertical successors, are the "molars" properly so called.

When the premolars and the molars are below their typical number, the absent teeth are missing from the forepart of the premolar series, and from the back part of the molar series. The most constant teeth are the fourth premolar and the first true molar; and, these being known by their order and mode of development, the homologies of the remaining molars and premolars are determined by counting the molars from before backwards, *e. g.* "one," "two," "three" and the premolars from behind forwards, *e. g.* "four," "three," "two," "one." The incisors are counted from the median line, commonly the foremost part of both upper and lower jaws, outwards and backwards. The first incisor of the right side is the homotype, transversely, of the contiguous incisor of the left side in the same jaw, and, vertically, of its opposing tooth in the opposite jaw ; and so with regard to the canines, premolars, and molars ; just as the right arm is the homotype of the left arm in its own segment, and also of the right leg of a succeeding segment. It suffices, therefore, to reckon and name the teeth of one side of either jaw in a species with the typical number and kinds of teeth ; *e. g.* the first, second, and third incisors, the first, second, third and fourth premolars ; the first, second, and third molars ; and of one side of both in jaws in any case.

The homologous teeth being thus determinable, they may be severally signified by a symbol as well as by a name. The incisors, *e. g.*, by their initial letter *i*, and individually by an added number, *i.* 1, *i.* 2, and *i.* 3; the canines by the letter *c.* ; the premolars by the letter *p.* ; and the molars by the letter *m.* ; these also being differentiated by added numerals. Thus, the number of these teeth, on each side of both jaws, in any given species, man *e. g.*, may be expressed by the following brief formula :—$i.\ \frac{2-2}{2-2},\ c.\ \frac{1-1}{1-1},\ p.\frac{2-2}{2-2},\ m.\ \frac{3-3}{3-3}=32$ ; and the homo logies of the individual teeth, in relation to the typical formula, may be signified by *i.* 1., *i.* 2.; *c.; p.* 3.; *m.* 1., *m.* 2., *m.* 3.: the suppressed teeth being *i.* 3., *p.* 1., and *p.* 2.

Examples of the typical dentition are exceptions in the actual creation but it was the rule in the forms of mammalia first introduced into this planet ; and that, too, whether the teeth were modified for animal or vegetable food. Fig. 33, *e. g.*, shows the dental series of the upper jaw of the *amphicyon major*, a mixed-feeding ferine animal, allied to the bear.

Figure 34 shows the dental series of the under jaw of a more strictly carnivorous beast, the *hyænodon ;* the fossil remains of a species of which have been discovered in the oldest

Fig. 33.

*Dentition of the Amphicyon major. Upper jaw.*

tertiary deposits of Hampshire. The symbols denote the homologies of the teeth. The true molars in the one are tuberculate, indicating its tendency to vegetable diet; in the other, they are carnassial, and betoken a peculiarly destructive and bloodthirsty species.

Fig. 34.

*Dentition of the Hyænodon. Lower jaw.*

In the Quarterly Geological Journal, No. 13, 1848, p. 36. pl. iv., I have described and figured the entire dental series of one side of the lower jaw of an extinct hoofed quadruped, the *dichodon cuspidatus*, from eocene or oldest tertiary strata also manifesting the normal number and kinds of teeth, but with such equality of height of crown, that no interspace is needed to lodge any of the teeth when the jaws are closed, and the series is as entire and uninterrupted as in the human subject. A great proportion of the upper jaw and teeth has been discovered, and the marks of abrasion on the lower teeth prove the series above to have been as entire and continuous as that below. The *anoplotherium*, from the gypsum quarries of Montmartre, geologically as ancient as the eocene clays of this island, long ago presented to Cuvier the same peculiar continuous dental series as is shown in the *dichodon*. In his original memoir, Cuvier described the canines as a fourth pair of incisors, on account of their small size and their trenchant shape; but he afterwards recognised their true homology with the larger and more laniariform canines of the *palæotherium*. The *chæropotamus*, the *anthracotherium*, the *hyopotamus*, the *hyracotherium*, the *oplotherium*, the *merycopotamus*, the *hippohyus*, and other ancient (eocene and miocene) tertiary mammalian genera presented the forty-four teeth, in number and kind according to that which is here propounded as the typical or normal dentition of the placental mammalia. Amongst the existing genera, the hog (*sus*) is one of the few that retain this type.

Fig. 35.

*Dentition of the Hog (Sus.)*

Figure 35, shows the entire permanent series, exposed in both jaws, and indicated individually by their symbols. Fig. 36 illustrates the phenomena of development which distinguish the premolars from the molars. The first premolar, *p.* 1, and

Fig. 36.

*Deciduous and permanent teeth* (Sus.)

the first molar *m.* 1, are in place and use, together with the three deciduous molars, *d.* 2, *d.* 3, and *d.* 4 ; the second molar, *m.* 2, has just begun to cut the gum ; *p.* 2, *p.* 3, and *p.* 4, together with *m.* 3, are more or less incomplete and concealed in their closed alveoli.

The premolars must displace deciduous molars in order to rise into place ; the molars have no such relations ; it will be observed, that the last deciduous molar, *d.* 4, has the same relative superiority of size to *d.* 3 and *d.* 2 which *m.* 3 bears to *m.* 2, and *m.* 1 ; and the crowns of *p.* 3 and *p.* 4 are of a more simple form than those of the milk-teeth which they are destined to succeed.

Teeth of each of the kinds above determined, and arbitrarily named "incisors," "canines," "premolars," "molars," have received other special names in regard to certain peculiarities of form or other property ; and the ablest comparative anatomists have been led astray in determining their homologies when they have suffered themselves to be guided exclusively by morphological characters. The premolars in the human subject have been called "bicuspids." The last upper premolar and the first lower true molar in the carnivora are termed, from their peculiar form, "sectorials," or "carnassial teeth," "molaires carnassières" of Cuvier. Teeth of and elongated conical form, projecting considerably beyond the rest, and of uninterrupted growth, are called "tusks ;" such are the incisors of the elephant and dugong, the canines of the boar and walrus : the long and large incisors of the rodents have been termed, from the shape and structure of their cutting edge, scalpriform or chisel-teeth, "*dentes scalpararii.*" The inferior incisors of the flying lemurs (*galeopithecus*) have the crown deeply notched like a comb, and are termed, "*dentes pectinati.*" The canines of the baboons are deeply grooved in front, like the poison-fangs "*dentes canaliculati,*" of some serpents. The compressed conical crowns of the molar teeth of the small clawed seals (*stenorhynchus*) are divided either like a trident, into three sharp points, or like a saw, into four or five points ; the molars of the great extinct *zeuglodon* had a similar form ; such teeth have been called *dentes serrati.* But the philosophical course of the knowledge of nature tends to explode needless terms of art, invented for unimportant varieties, and to establish and fix the meaning of those terms that are the signs of determinate species of things.

The Cuviers divided the molar series of teeth, according to their form, into three kinds : "false molars," "carnassials," and "tubercular molars ;" and, in giving the generic characters of mammalia, based the dental formulæ on this system : thus the genus *felis* is characterised as having "fauses molaires $\frac{2}{2}$—$\frac{2}{2}$, carnassières $\frac{1}{1}$—$\frac{1}{1}$, tuberculeuses $\frac{1}{1}$—$\frac{1}{1}$; $=\frac{8}{6}$."

The uninterrupted line marked "Cuvier" in V. felis of fig. 37, intersects the teeth in each jaw called called carnassières ; those anterior to them being the teeth called "fausses molaires ;" the single tooth behind in the upper jaw is the "tuberculeuse." Most zoologists, both at home and abroad, have adopted the Cuvierian system of formalising the molar teeth. It seems a very natural one in the case of the cat genus ; the tooth *p.* 4 above plays upon that, *m.* 1, below, which has a similar remarkable carnassial modification of form ; they fit, indeed, almost as Cuvier describe, like the blades of a pair of

Fig. 37.

*Homologies of the teeth in Diphyodont Mammals.*

*Cuvier. De Bl.*

scissors; the two teeth in advance of the carnassial in the upper jaw (*p.* 3, *p.* 2) in like manner are opposed to the same number of "fausses molaires" (*p.* 4, *p.* 3) in the under jaw, and the canine *c.* above plays upon the canine below; all seems straightforward and symmetrical, save that the little tubercular, *m.* 1, above has no opponent in the lower jaw. And, perhaps, the close observer might notice that whilst the upper canine, *c.*, glides behind its homotype below, the first upper false molar (*p.* 2) passes anterior to the crown of the first false molar (*p.* 3) below; and that the second false molar and carnassial of the upper jaw are also a little in advance of those teeth in the under jaw when the mouth is shut.

In passing to the dentition of the dog (fig. 37, canis,) formulised by Cuvier, as: "fausses molaires $\frac{3}{4}-\frac{3}{4}$, carnassières $\frac{1}{1}-\frac{1}{1}$, tuberculeuses $\frac{2}{2}-\frac{2}{2} = \frac{1}{1}\frac{2}{4}$ it will be observed, that here the first upper false molar (*p.* 1,) differs from that in *felis*, inasmuch, as when the mouth is shut, it preserves the same relative position to its opponent below (*p.* 1,) which the upper canine does to the lower canine, and that the same may be said of the second and the third false molars; but that with regard to the carnassial above (*p.* 4,) this tooth repeats the same relative position in regard to the fourth false molar below (*p.* 4,) and not to that tooth, *m.* 1, which Cuvier regarded as the lower homotype of the carnassial; and, indeed, the more backward position of the lower carnassial is so slight, that its significance might well be overlooked, more especially, as the two succeeding tubercular teeth above were opposed to two similar tuberculars below. Cuvier, therefore, leaves us to conclude, that the tooth which had no homotype or answerable opponent above, was either the fourth "fausse molaire" below, or else the first. How unimportant size and shape are, and how significant relative position is in the determination of the homologies of teeth as of other parts, may be learnt before quitting the natural order of carnivora; *e. g.* by the condition of the dental system in the bear (fig. 37, ii. Ursus.) Here the lower tooth, *m.* 1, instead of presenting the carnassial character and resembling in form the upper tooth, (*p.* 4,) which is the homologue of the upper carnassial in the dog, has a tubercular crown, and corresponds in size as well as shape with the upper tooth *m.* 1, to which it is almost wholly opposed, and with the same slight advance of position which we observe in the lower canine as compared with the upper one, and in the four lower premolars, (*p.* 1, *p.* 2, *p.* 3, *p.* 4,) as compared with their veritable homotypes above. F. Cuvier divides the molar series of the genus *ursus* into "fausses molaires, $\frac{3-3}{4-4}$, carnassières $\frac{1-1}{1-1}$, tuberculeuses $\frac{2-2}{2-2} = \frac{12}{14}$." The tendency in every thinker to generalise and to recognize nature's harmonies, has led him here to use the term "carnassière" in an arbitrary sense, and to apply it to a tooth above (*p.* 4,) which he owns has such a shape and diminished size, as would have led him to regard it as merely a false molar, but, that the upper carnassial would then have entirely disappeared; and it has also led him to give the name "carnassière" to a tooth below, *m.* 1, which he, nevertheless, describes as having a tubercular and not a trenchant crown. In so natural a group as the true carnivora, it was impossible to overlook the homologues of the trenchant carnassials of the lion, even when they had become tubercular in the omnivorous bear; and Cuvier, therefore, having determined and defined the teeth, so called in the feline genus, felt compelled to distinguish them by the same names after they had lost their specific formal character. And if, indeed, he had succeeded in discovering the teeth which were truly answerable or homotypal in the upper and lower jaws, the term "carnassial" might have been retained as an arbitrary one for such teeth, and have been applied to their homologues in man, the ruminant, or the pachyderm, where they are as certainly determinable as in those aberrant carnivores, in which they have equally lost their sectorial shape. But the inconvenience of names indicative of such specialties of form will be very obvious when the term "tuberculeuses" comes to be applied to the three hindmost teeth in the *hyænodon*, (fig. 34,) which teeth answer to the broad crushing teeth, *m.* 1, *m.* 2, and *m.* 3, in the bear and some other existing carnivora. The analogous term "molar," having a less direct or descriptive meaning, is therefore, so much the better as the requisite arbitrary name of a determinate species of teeth.

Had Cuvier been guided in his determinations of the teeth by their mutual opposition in the closed mouth, and had studied them with this view in the carnivora, with the dentition most nearly approaching to the typical formula, viz. the bear, he could then have seen that the three small and inconstant lower premolars (*p.* 1, *p.* 2, *p.* 3,) were the homotypes of the three small and similarly inconstant premolars above; that the fourth false molar (*p.* 4) below, which, as he observes, "alone has the normal form," was truly the homotype of the tooth above, (*p.* 4,) which he found himself compelled to reject from the class of "fausses molaires," notwithstanding it presented their normal form; that the turbercular tooth, *m.* 1, which he calls "carnassière" in the lower jaw, was the veritable homotype of his first "molaire tuberculeuse" above (*m.* 1,) and that the tooth in the inferior series which had no answerable one above was his second "tuberculeuse," (my *m.* 3,) and not any of the four false molars. The true second tubercular above (*m.* 2) is, however, so much developed in the bear as to oppose both *m.* 2 and *m.* 3 in the lower jaw, and it might seem to include the homotypes of both those teeth coalesced. One sees with an interest such as only these homological researches could excite, that they were distinctly developed in the ancient *amphicyon*, (fig. 33,) which accordingly presents the typical formula. Thus, I repeat, the study of the relative position of the teeth of the bear might have led to the recognition of their real nature and homologies, and have helped to raise the mask of their extreme formal modifications, by which they are adapted to the habits of the more blood-thirsty carnivora. But the truth is plainly and satisfactorily revealed, when we come to trace the course of development and succession of these teeth. The weight which must ever attach itself to an opinion sanctioned by the authority of both the Cuviers, demands, that a conclusion contrary to theirs, and which seems to be opposed by nature herself, in certain instances, should be supported by all the evidence of which such conclusion is susceptible.

I proceed, therefore, to show how, in the bear, my determinations of the teeth are established by their development, as well as by their relative position. As the question only concerns the molar series, the remarks will be confined to these. In the jaws of the young bear, figured in cut 38, the first pre-

Fig. 38.

*Deciduous and permanent dentition of the Bear* (Ursus.)

molar, *p.* 1, is the only one of the permanent series in place; the other grinders in use, are the deciduous molars, *d.* 2, *d.* 3, and *d.* 4; *d.* 2 will be displaced by *p.* 2, *d.* 3 by *p.* 3, and *d.* 4, by the tooth, *p.* 4, which, notwithstanding its size and shape, Cuvier felt himself compelled to discard from the series of false molars; but which we now see, is proved by its developmental relations to *d.* 4, as well as by its relative position and similarity to *p.* 4 in the lower jaw, (fig. 37, Ursus,) to be veritably the last of the premolar series, and to agree not in shape only, but in every essential character, with the three preceding teeth called by Cuvier "fausses molaires." So, likewise, in the lower jaw, we see, that the primitive deciduous series, *d.* 1, *d.* 2, *d.* 3 and *d.* 4, will be displaced by the corresponding premolars, *p.* 1, *p.* 2, *p.* 3 and *p.* 4; and that the tooth *m.* 1, called carnassière by Cuvier, in the lower jaw, differs essentially from that *p.* 4, so called in the upper jaw by being developed without any vertical predecessor or deciduous tooth.

The same law of development and succession prevails in the genus *canis* (fig. 38.) Although the tooth *m.* 1, in the lower jaw, has exchanged the tubercular for the carnassial form, it is still developed, as in the bear, behind the deciduous series, and independently of any vertical predecessor; and the tooth *p.* 4 above, although acquiring a relative superiority of size to its homologue in the bear, and more decidedly a carnassial form, is not the homotype of the permanent carnassial below, but of that premolar (*p.* 4,) which is destined to displace the deciduous carnassial, *d.* 4. The symbols sufficiently indicate the relations of the other teeth, and the conclusions that are to be drawn from them as to their homologies. It is interesting to observe in the deciduous, as well as in the permanent series, that the lower carnassial *d.* 4, is not the homotype of the upper one, *d.* 3, but of the tooth which Cuvier calls the "tuberculeuse du lait," *d.* 4 in the upper jaw.

In the genus *felis*, (fig. 37,) the small permanent tubercular molar of the upper jaw, *m.* 1, has cut the gum before its analogue *d.* 3, of the deciduous series has been shed ; but though analogous in function, this is not homologous with, or the precedent tooth to *m.* 1 ; but, as in the dog, to the great carnassially modified premolar, *p.* 4. In the lower jaw the tooth (*m.* 1,) which is functionally analogous to the carnassial above, is also, as in the dog, the first of the true molar series, and the homotype of the little tubercular tooth (*m.* 1) above. And the homologies of the permanent teeth *p.* 4 above, and *m.* 1 below, with those so symbolised in the dog, (fig. 39,) teach us that the teeth which are wanting, in order to equal the number of those in the canine dentition, are *m.* 2 in the upper jaw, *m.* 2 and *m.* 3 in the lower jaw ; *p.* 1 in the upper jaw, *p.* 1 and *p.* 2 in the lower jaw ; thus illustrating the rule enuntiated above, that, when the molar series falls short of the typical number, it is from the two extremes of such series that the teeth are taken, and that so much of the series as is retained, is thus preserved unbroken. In the great extinct sabre-toothed tiger (*machairo-*

Fig. 39.

*Deciduous and permanent teeth in the Dog* (Canis.)

*dus*, fig. 37, vi,) the series is still further reduced by the loss of *p.* 2 in the upper jaw.

That the student may test for himself the demonstration which the developmental characters above defined, yield of the true nature and homologies of the feline dentition—the most modified of all in the terrestrial carnivora, he is recommended to compare with nature the following details of the appearance and formation of the teeth in the common cat. In this species, the deciduous incisors *d. i.* begin to appear between two and three weeks old; the canines *d. c.* next, and then the molars *d. m.* follow, the whole being in place before the sixth week. After the seventh month they begin to fall in the same order; but the lower sectorial molar *m.* 1, and its tubercular homotype above (*m.* 1) appear before *d.* 2, *d.* 3, and *d.* 4 fall. The longitudinal grooves are very faintly marked in the deciduous canines. The first deciduous molar (*d.* 2,) in the upper jaw, is

Fig. 40.

*Deciduous and permanent teeth in the Lion* (Felis.)

a very small and simple one-fanged tooth; it is succeeded by the corresponding tooth of the permanent series, which answers to the second premolar (*p.* 2) of the hyæna and dog. The second deciduous molar (*d.* 3) is the sectorial tooth; its blade is trilobate, but both the anterior and posterior smaller lobes are notched, and the internal tubercle, which is relatively larger than in the permanent sectorial, is continued from the base of the middle lobe, as in the deciduous sectorial of the dog and hyæna; it thus typifies the form of the upper sectorial, which is retained in the permanent dentition of several viverrine and musteline species. The third or internal fang of the deciduous sectorial, is continued from the inner tubercle, and is opposite the interspace of the two outer fangs. The musteline type is further adhered to by the young feline in the large proportional size of its deciduous tubercular tooth *d.* 4. In the lower jaw, the first milk-molar (*d.* 3) is succeeded by a tooth (*p.* 3) which answers to the third lower premolar in the dog and civet. The deciduous sectorial (*d.* 4,) which is succeeded by the premolar (*p.* 4,) answering to the fourth in the dog, has a smaller proportional anterior lobe, and a larger posterior talon, which is usually notched; thereby approaching the form of the permanent lower sectorial tooth in the *mustelidæ.*

In the article carnivora, (vol. i, p. 488,) the remarks on the teeth are limited chiefly to their physiological adaptations. A description of some of their more remarkable structures will here be given, according to the idea of the nature of the teeth above developed. The dental formula of the dog, jackal, wolf, and fox, is illustrated in fig. 37, iii, canis.

In the *megalotis*, or long-eared fox, (*otocyon*, licht.,) the deviation from the typical dentition of the *canidæ* is effected by excess of development; two additional true molars being present on each side of the upper, and one on each side of the lower jaw, in the permanent series of teeth; and an approach is made by the modified form of the sectorial molar, and of some of the other teeth, to the dentition of *viverridæ.* This family of carnivora, which comprehends the civets, genets, ichneumons, musangs, surikates, and mangues, is characterized, with few exceptions, by the following formula:—*i.* $\frac{3-3}{3-3}$; *c.* $\frac{1-1}{1-1}$; *p.* $\frac{4-4}{4-4}$; *m.* $\frac{2-2}{2-2}$: $= 40$. It differs from that of the genus *canis* by the absence of a tubercular tooth (*m.* 3) on each side of the lower jaw; but, in thus making a nearer step to the typical carnivorous dentition, the *viverridæ*, on the other hand, recede from it by the less trenchant and more tubercular character of the sectorial teeth.

The canines are more feeble, and their crowns are almost smooth; the premolars, however, assume a formidable size and shape in some aquatic species, as those of the sub-genus *cynogale*, in which their crowns are large, compressed, triangular, sharp-pointed, with trenchant and serrated edges, like the teeth of certain sharks, (whence the name *squalodon*, proposed for one of the species,) and well adapted to the exigencies of quadrupeds, subsisting principally on fish: the opposite or obtuse, thick form of the premolars is manifested by some of the musangs, as *paradoxurus auratus*. The upper sectorial tooth, *p.* 4, is characterized by having its inner tubercle larger, the middle conical division of the blade thicker, and the posterior one smaller than in the genus *canis*. This tooth advances to beneath the ant-orbital foramen in the musangs (*paradoxurus*:) it is situated farther back in the civets and genets, in which the blade of the sectorial is sharper. This shows that relative position to the zygomatic or molar process of the maxillary is not a good character.

In the lower jaw the sectorial tooth (*m.* 1) manifests its true molar character by the presence of an additional pointed lobe on the inner side of the two lobes forming the blade at the forepart of the crown: the posterior, low, and large lobe of the tooth being also tri-tuberculate, as in the dog. The last molar (*m.* 2) has an oval crown, with four small tubercles, resembling the penultimate lower molar in the dog, with which it corresponds.

The deciduous dentition consists, in the viverrine family, of: incisors $\frac{3-3}{3-3}$; canines $\frac{1-1}{1-1}$; molars $\frac{3-3}{3-3}$ :=28. If the first permanent premolar has any predecessor, it must be rudimental, and disappear early in both jaws; the second premolar displaces the first normally developed deciduous molar; the third upper premolar displaces and succeeds the deciduous sectorial, which has a sharper and more compressed blade, and a relatively smaller internal tubercle, than the permanent sectorial. This tooth displaces the last deciduous molar, which is a tubercular tooth, resembling, in form, the first of the two upper permanent tuberculars; these coming into place without pushing out any predecessors, enter into the category of true molar teeth. In the lower jaw the third premolar displaces the deciduous sectorial, which has three trenchant lobes and a relatively smaller posterior talon than the permanent sectorial. The fourth premolar displaces the third or tubercular milk-molar. The permanent sectorial and tubercular molars, displace no predecessors, and are therefore *m.* 1 and *m.* 2.

The first premolar, *p.* 1, is not developed at any period in the mangues (*crosarchus*,) the suricates (*ryzæna*,) or the *mangusta paludinosa;* these viverrines, therefore, retain throughout life, more of the immature characters of the family, and in the same degree approach, in the numerical characters of their dentition, to the more typical carnivora.

The alternate interlocking of the crowns of the teeth of the upper and lower jaws, which is their general relative position in the carnivora, is well marked in regard to the premolars of the *viverridæ*, fig 37, iv :) as the lower canine is in front of the upper, so the first lower premolar rises into the space between the upper canine and first upper premolar; the fourth lower premolar, in like manner, fills the space between the third upper premolar (*p.* 3) and the sectorial tooth (*p.* 4,) playing upon the anterior lobe of the blade of that tooth, which indicates by its position, as by its mode of succession, that it is the fourth premolar of the upper jaw. The first true molar below, modified as usual in the *carnivora* to form the lower sectorial, sends the three tubercles of its anterior part to fill the space between the sectorial (*p.* 4) and the first true molar (*m* 1) above. In the musangs, the lower sectorial is in more direct opposition to its true homotype, the first tubercular molar in the upper jaw; and these Indian *viverridæ* (*paradoxuri*) are the least carnivorous of their family, their chief food consisting of the fruit of palm-trees, whence they have been called "palm-cats."

*Hyæna.*—The dentition of this genus presents a nearer approach to the strictly carnivorous type, by the reduction of the tubercular to a single minute tooth on each side of the upper jaw, the inferior molars being all conical or sectorial teeth: the molar teeth in both jaws are larger and stronger, and the canines smaller in proportion than in the feline species, from the formula of which the dentition of the hyæna differs numerically only in the retention of an additional premolar tooth, *p.* 1 above and *p* 2 below, on each side of both jaws. The dental formula of the genus *hyæna* is:—*in.* $\frac{3-3}{3-3}$, *c.* $\frac{1-1}{1-1}$, *pm.* $\frac{4-4}{3-3}$, *m.* $\frac{1-1}{1-1}$ := 34. The crowns of the incisors form almost a straight transverse line in both jaws, the exterior ones, above, being much larger than the four middle ones, and extending their long and thick inserted base further back : the crown of the upper and outer incisor (*i.* 3,) is strong, conical, recurved, like that of a small canine, with an anterior and posterior edge, and a slight ridge along the inner side of the base. The four intermediate small incisors have their crown divided by a transverse cleft into a strong anterior, conical lobe, and a posterior ridge, which is notched vertically; giving the crown the figure of a trefoil. The lower incisors gradually increase, in size, from the first to the third; this and the second have the crown indented externally; but they have not the posterior notched ridge like the small upper incisors; the apex of their conical crown fits into the interspace of the three lobes of the incisor above. The canines have a smooth convex exterior surface, divided by an anterior and posterior edge from a less convex inner side : this surface is almost flat, and of less relative extent in the inferior canines. The first premolar above (*p.* 1) is very small, with a low, thick, conical crown : the second presents a sudden increase of size, and an addition of a posterior and internal basal ridge to the strong cone. The third premolar exhibits the same form on a still larger scale, and is remarkable for its great strength. The posterior part of the cone of each of these premolars is traversed by a longitudinal ridge. The fourth premolar is the carnassial tooth, and has its long blade divided by two notches into three lobes, the first a small thick cone, the second a long and compressed cone, the third a horizontal sinuous trenchant plate: a strong triedral tubercle is developed from the inner side of the base of the anterior part of the crown. The single true molar of the upper jaw (*m.* 1) is a tubercular tooth of small size: transversely oblong in the *hyæna vulgaris* and *H. fusca;* smaller and sub-circular in the *hyæna crocuta;* still smaller and implanted by a single fang in the *hyæna spelæa:* in all the existing species of hyæna it has two fangs. The first premolar of the lower jaw (*p.* 2) fits into the interspace between the first and second premolars above, and answers, therefore, to the second lower premolar in the *viverridæ:* it is, accordingly, much larger than the first (*p.* 1) above; it has a ridge in the forepart of its cone, and a broad basal talon behind. The second (*p.* 3) is the largest of the lower premolars, has an anterior and a posterior basal ridge, with a vertical ridge ascending upon the fore as well as the back part of the strong rounded cone: the third premolar (*p.* 4,) is proportionably less in the *hyæna crocuta* than in the *H. vulgaris:* its posterior ridge is developed into a small cone; the last tooth (*m.* 1) is the sectorial, and consists almost entirely of a blade divided by a vertical fissure into two sub-equal compressed pointed lobes: the points are less produced than in the felines, but the lower sectorial of the hyæna is better distinguished by the small posterior basal talon, from which a ridge is continued along the inner side of the base, and is slightly thickened at the forepart of the crown. According to the relative position of the crowns of the premolars, the third below ought to be the last, being analogous to the fourth in the *viverridæ*, and the sectorial should be first true molar: we shall find this view confirmed by the test of the mode of succession of the permanent teeth. But the mode of implantation of the premolar and molar teeth may first be noticed. The first upper premolar has but one fang; the second and third have each two; the sectorial tooth has three, the two anterior ones on the same transverse line, the inner one supporting the tubercle. The lower premolars and sectorial have each two fangs, there being none truly answering to the first above: the anterior root of the lower (*p.* 1) sectorial tooth is very strongly developed in the great extinct cave-hyæna.

The deciduous teeth consist of:—*i.* $\frac{3-3}{3-3}$, *c.* $\frac{1-1}{1-1}$, *m.* $\frac{3-3}{3-3}$,= 28. The figure of the skull of the young *hyæna crocuta* in the posthumous edition of the "Ossemens Fossiles," 8vo. 1836, pl. 190, fig. 3, shows that stage when the correspondence with the formula of the genus *felis* is completed by the appearance, in the upper jaw, of a small premolar in the interspace between the canine and first molar of the deciduous series: but this appearance is due to the apex of the first permanent premolar which cuts the gum before any of the normal deciduous teeth are shed : whether it is preceded, as in the dog, by a deciduous germ-tooth in the fetus, I know not. The first normal deciduous molar is two-fanged, and has a more compressed, and consequently more carnivorous crown than that of the second permanent premolar by which it is succeeded. The second deciduous molar is the sectorial tooth: the inner tubercle is continued from the base of the middle lobe, and thus resembles the permanent sectorial of the glutton (*gulo*) and many other *mustelidæ ;* the deciduous tubercular molar is relatively larger than in the adult *hyæna,* and offers another feature of resemblance to the permanent dentition of the glutton. It is also worthy of remark, that the exterior incisor of the upper jaw is not only absolutely, but relatively smaller in the immature than in the adult dentition of the hyæna, and again illustrates the resemblance to the more common type of dentition in the carnivora.

The first and second deciduous molars below, have more compressed conical crowns than their successors; the third deciduous molar is the sectorial tooth, and again, as in *gulo,* has a better developed hinder tubercle than the permanent sectorial; it is not displaced by this tooth, but, as in other carnivora, by a premolar of more simple character. The permanent sectorial is developed posteriorly, and rises, like other true molars, without displacing a deciduous predecessor.

The permanent dentition of the *hyæna,* as of other genera or families of the carnivora, assumes those characteristics which adapt it for the peculiar food and habits of the adult, and mark the deviation from the common type, which always accompanies the progress to maturity. The most characteristic modification of this dentition, is the great size and strength of the molars as compared with the canines ; and more especially, the thick and strong conical crowns of the second and third premolars in both jaws, the base of the cone being belted by a strong ridge which defends the subjacent gum.* This form of

* An eminent civil engineer, to whom I showed the jaw of a hyæna, observed that the strong conical tooth with its basal ridge, was a perfect model of a hammer for breaking stones for roads.

tooth is especially adapted for gnawing and breaking bones, and the whole cranium has its shape modified by the enormous development of the muscles which work the jaws and teeth in this operation.* Adapted to obtain its food from the coarser parts of animals which are left by the nobler beasts of prey, the hyæna chiefly seeks the dead carcass, and bears the same relation to the lion which the vulture does to the eagle. In consequence of the quantity of bones which enter into its food, the excrements consist of solid balls of a yellowish white color, and of a compact earthy fracture. Such specimens of the substance, known in the old *materia medica*, by the name of "album græcum," were discovered by Dr. Buckland, in the celebrated ossiferous cavern at Kirkdale. They were recognised at first sight, by the keeper of a menagerie, to whom they were shown, as resembling, both in form and appearance, the fœces of the spotted hyæna; and, being analysed by Dr. Wollaston, were found to be composed of the ingredients that might be expected in fœcal matter derived from bones, viz. phosphate of lime, carbonate of lime, and a very small proportion of the triple phosphate of ammonia and magnesia. This discovery of the coprolites of the hyæna formed, perhaps, the strongest of the links in that chain of evidence, by which Dr. Buckland proved, that the cave at Kirkdale, in Yorkshire, had been, during a long succession of years, inhabited as a den by hyænas, and that they dragged into its recesses the other animal bodies, whose remains splintered, and bearing marks of the teeth of the hyæna, were found mixed indiscriminately with their own.—*By Professor Owen, from the Cyclopœdia of Anatomy and Physiology.*

* "The strength of the hyæna's jaws is such, that, in attacking a dog, he begins by biting off his leg at a single snap." Buckland, "Reliquiæ Diluvianæ," p. 23.

## Figures and Tables

**Fig. 29 f1:**
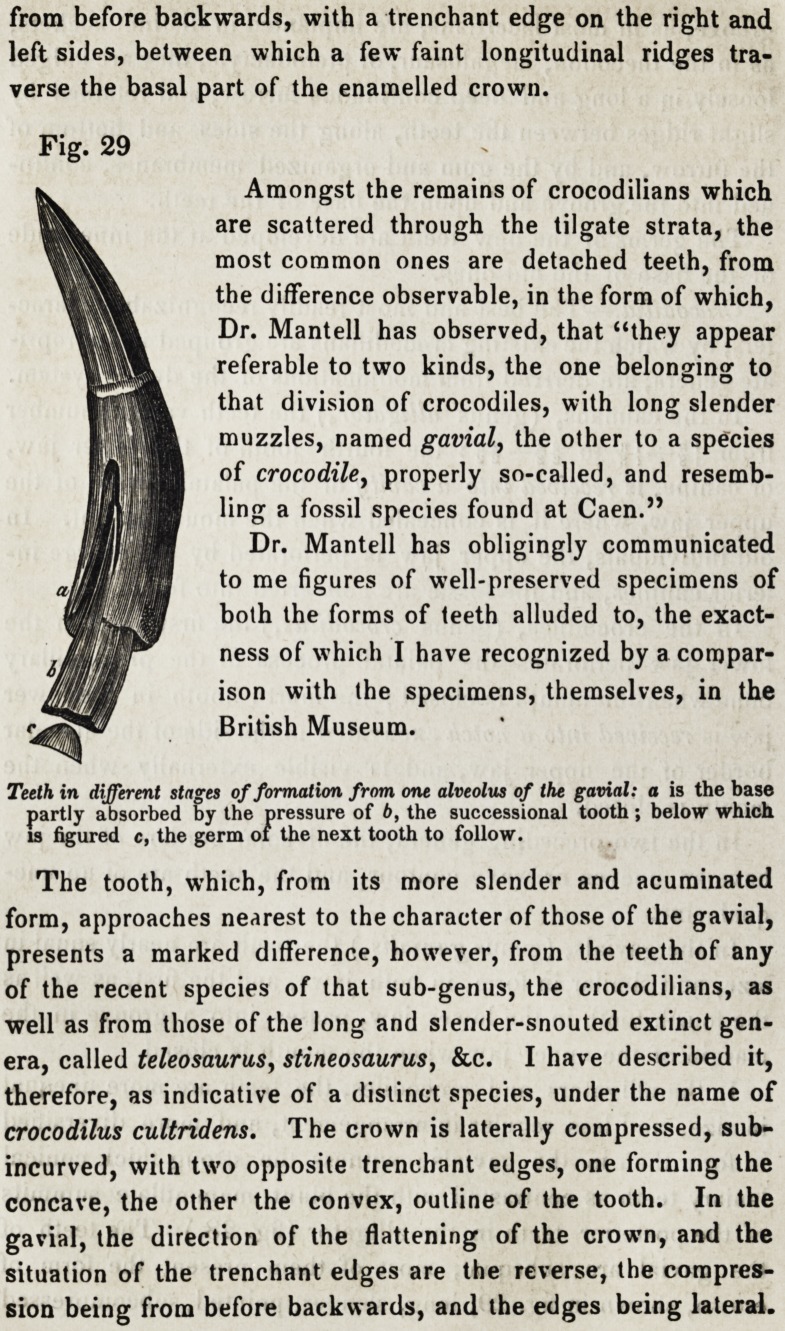


**Fig. 30. f2:**
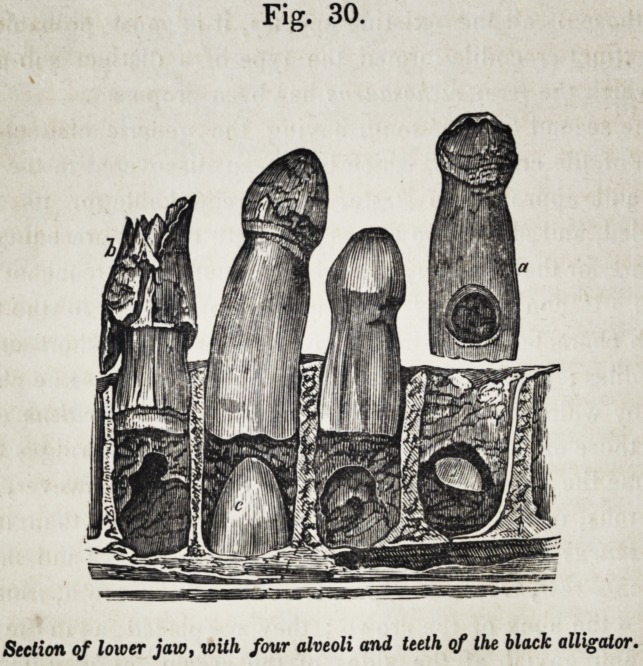


**Fig. 31. f3:**
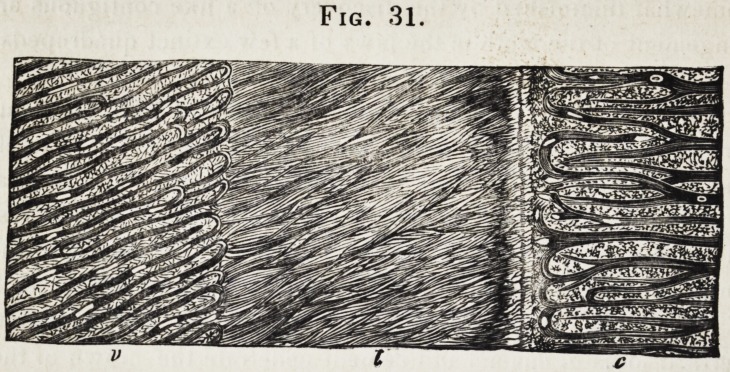


**Fig. 32. f4:**
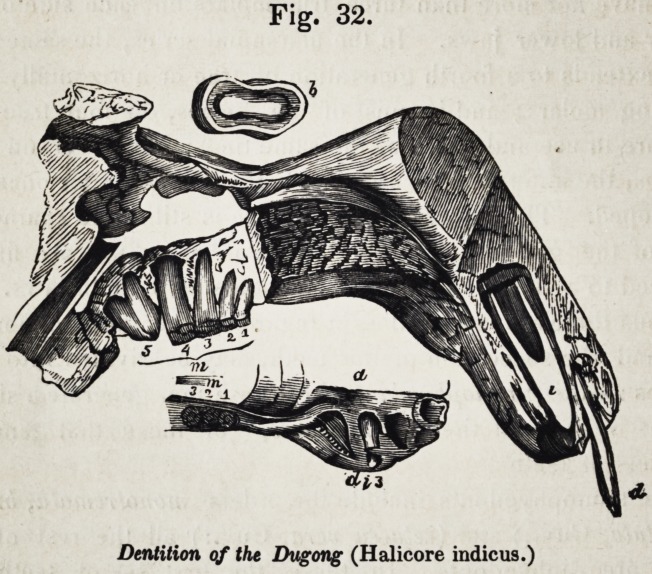


**Fig. 33. f5:**
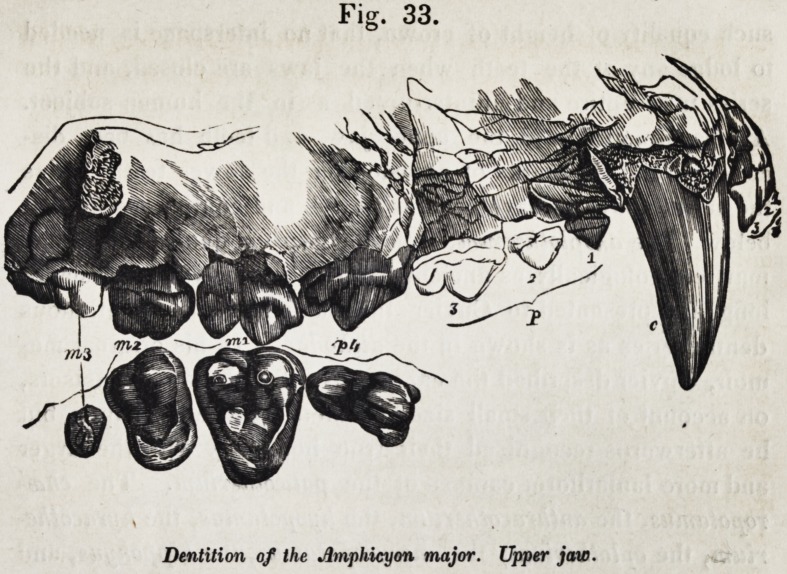


**Fig. 34. f6:**
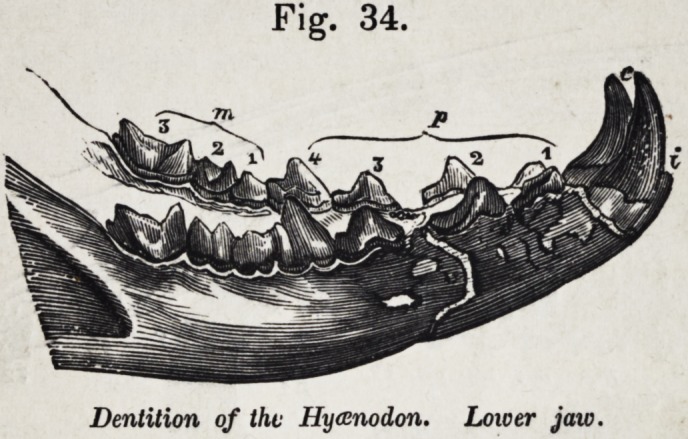


**Fig. 35. f7:**
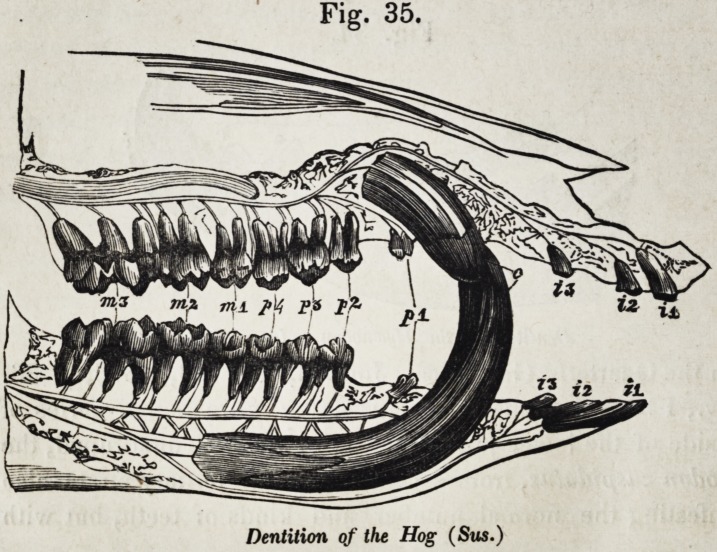


**Fig. 36. f8:**
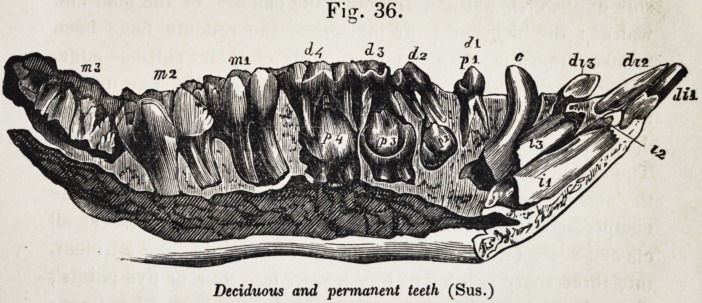


**Fig. 37. f9:**
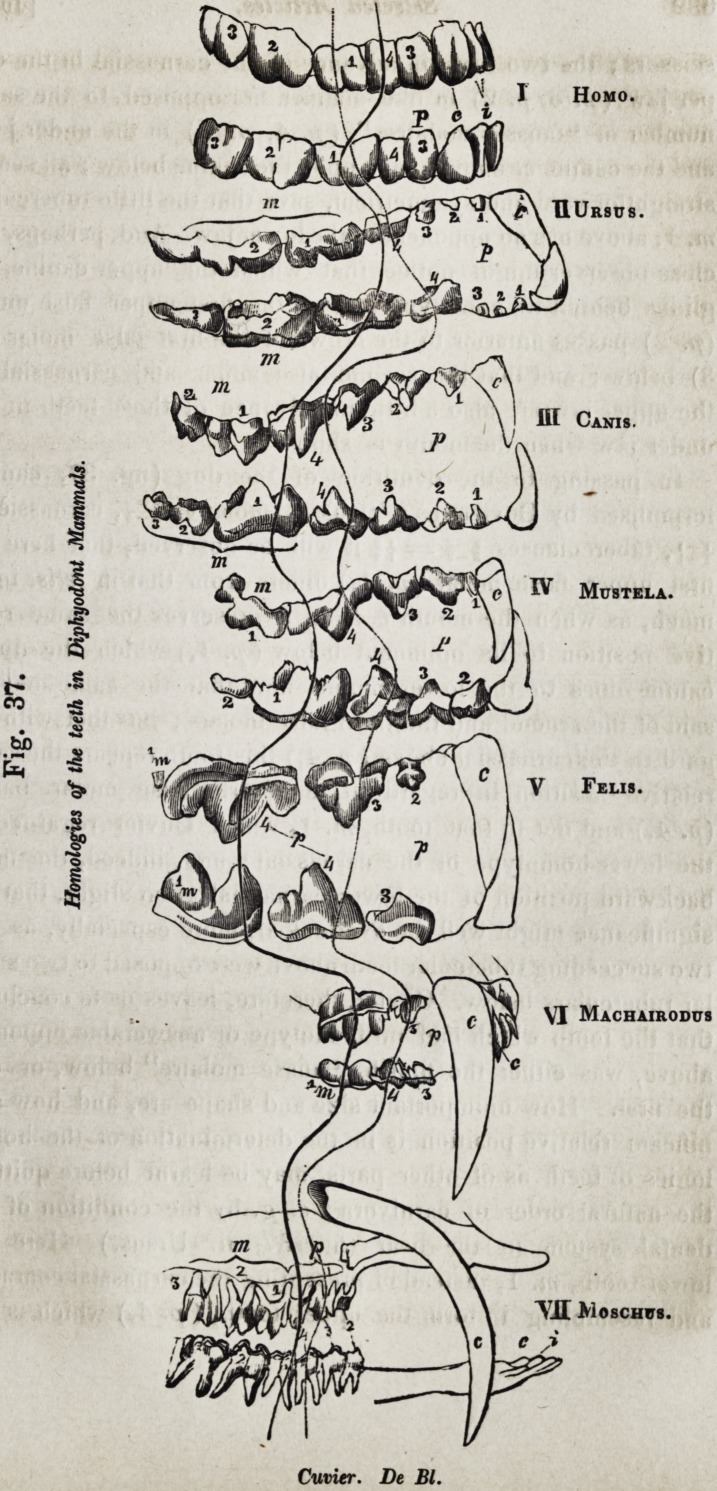


**Fig. 38. f10:**
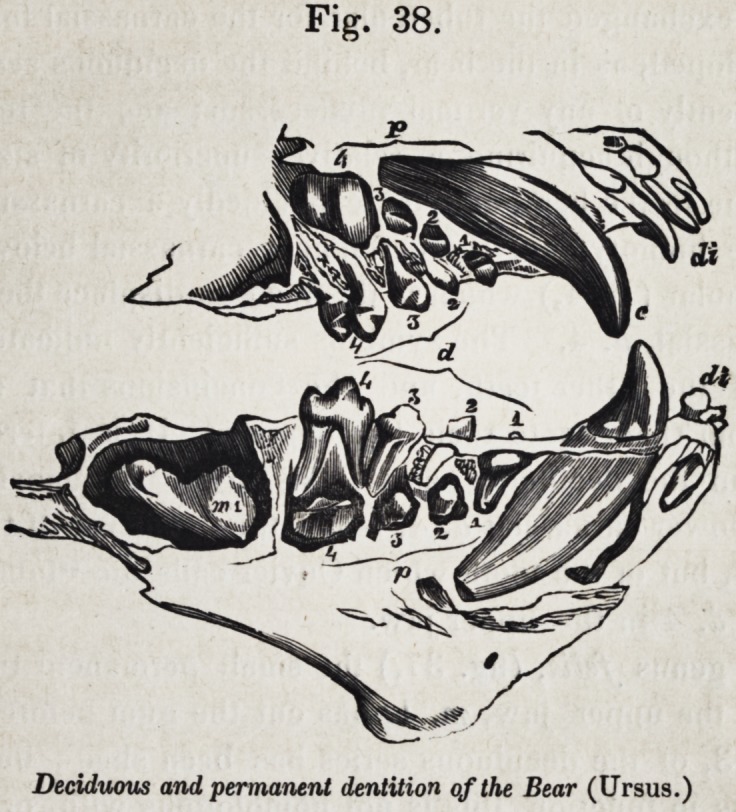


**Fig. 39. f11:**
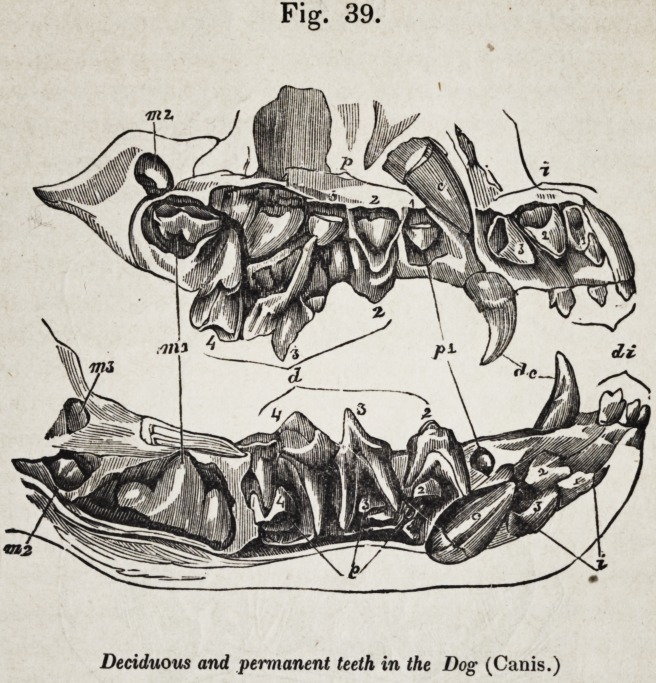


**Fig. 40. f12:**